# Identification of potential biomarkers of long non-coding RNAs in neuropathic pain using bioinformatic analysis: A protocol for systematic review and meta-analysis

**DOI:** 10.1097/MD.0000000000025147

**Published:** 2021-03-26

**Authors:** Yongzhi Fan, Na Li, Xianbao Yao

**Affiliations:** Department of Pain Management, Central Hospital of Wuhan, Tongji Medical College, Huazhong University of Science and Technology, Wuhan, Hubei Provine, China.

**Keywords:** long non-coding RNA, meta-analysis, neuropathic pain, protocol

## Abstract

**Background:**

Long non-coding RNAs (LncRNAs) play important roles in the regulation of neuropathic pain (NP) development. LncRNAs dysregulations are related to the development of NP. However, a comprehensive meta-analysis has never been conducted to assess the relationship between LncRNAs and NP. To combine the results of dysregulated LncRNAs in individual NP studies and to identify potential LncRNAs biomarkers.

**Methods:**

LncRNAs profiling studies of NP were extracted from Pubmed, Web of science, Embase, Google Scholar, and Chinese National Knowledge Infrastructure, and the Chinese Biomedical Literature Database if they met the inclusion criteria. The meta-analysis was conducted using a random effects model to identify the effect of each multiple-reported LncRNAs. We also performed subgroup analysis according to LncRNAs detecting methods and sample type. Sensitivity analysis was performed on the sample size. Bioinformatic analysis was performed to identify the potential biomatic functions. All results were represented as log10 odds ratios.

**Results:**

This review will be disseminated in print by peer-review.

**Conclusion:**

The identified LncRNAs may be closely linked with NP and may act as potentially useful biomarkers.

**Ethics and dissemination:**

The private information from individuals will not publish. This systematic review also will not involve endangering participant rights. Ethical approval is not available. The results may be published in a peer- reviewed journal or disseminated in relevant conferences

**OSF REGISTRATION NUMBER:**

DOI 10.17605/OSF.IO/ZRX7C.

## 1 Introduction

Neuropathic pain (NP) is 1 of the most common chronic pain in humans, and is characterized by increased responsiveness of nociceptive neurons in the peripheral and central nervous system.^[1–3]^ Peripheral and central sensitization represent changes in the functional state of nociceptive neurons and are caused by changes in a large number of functional proteins and signal pathways in neurons and glial cells.^[4]^ NP is a refractory chronic disease that seriously endangers the life quality of patients and brings heavy burden to society and families.^[5,6]^ The whole world spends hundreds of millions of dollars on the treatment of chronic pain every year. However, at present, conventional analgesic drugs are difficult to achieve the ideal analgesic effect.^[7–9]^ Therefore, there is an urgent need to develop new targets for the treatment of NP.

Long non-coding RNAs (LncRNAs) are a new non-coding protein RNA with a transcript of more than 200 bp.^[10]^ Although LncRNAs were initially considered as a transcriptional by-product, recent advances in technology provided a new understanding of their important role in gene regulation and disease pathogenesis.^[11,12]^ Recent data display that LncRNAs can participate in gene expression by regulating transcription, post-transcriptional processing, chromatin remodeling and the production of small non-coding RNA.^[13–15]^ Existing evidence reveal that neuro-inflammation is characterized by glial cell activation, leukocyte infiltration and the production of inflammatory mediators, which plays an important role in the pathogenesis of NP.^[16,17]^ LncRNAs are sianificant in regulating the activation of nerve cell surface receptors, the transcription of related proteins and the release of inflammatory factors.^[16–18]^ Consequently, meta-analysis is essential to determine the valuable LncRNAs biomarkers and potential therapeutic targets for NP.

So far, no reliable evaluation system has been observed for the relationship between LncRNAs expression and NP. However, there is no systematic comment on the evidence, and the relationship between the expression of LncRNAs and NP is still not fully understood. The objective of this study was to summarize and manage all differentially expressed LncRNAs presented in current NP studies and then conduct a meta-analysis to identify the potential biomarkers of consistently dysregulated LncRNAs which had been shown in reproducible NP studies.

## 2 Methods

### 2.1 Study registration

The protocol of the systematic review has been registered on Open Science Framework, and the registration number is DOI 10.17605/OSF.IO/ZRX7C. This meta-analysis protocol is based on the Preferred Reporting Items for Systematic Reviews and meta-analysis (PRISMA) Protocols statement guidelines.^[19]^

### 2.2 Data sources and search strategy

The following electronic bibliographic databases are searched to identify relevant studies: Pubmed, Web of science, Embase, Google Scholar, and Chinese National Knowledge Infrastructure, and the Chinese Biomedical Literature, up to December 2020. The combination of Medical Subject Headings words and free words is used in the search. References of the literature are also included. According to different characteristics of the database, the retrieval strategy of title, abstract or keyword is adjusted. The language is limited to Chinese and English. The search terms are illustrated in Table 1.

**Table 1:** Search strategy in PubMed database.

Number	Search terms
#1	Neuralgia[MeSH]
#2	Nerve Pain[Title/Abstract]
#3	Neurodynia[Title/Abstract]
#4	Paroxysmal Nerve Pain[Title/Abstract]
#5	Neuralgia, Atypical[Title/Abstract]
#6	Neuralgia, Iliohypogastric Nerve[Title/Abstract]
#7	Neuralgia, Ilioinguinal[Title/Abstract]
#8	Neuralgia, Perineal[Title/Abstract]
#9	Neuralgia, Stump[Title/Abstract]
#10	Neuralgia, Supraorbital[Title/Abstract]
#11	Neuralgia, Vidian[Title/Abstract]
#12	Neuropathic Pain[Title/Abstract]
#13	Atypical Neuralgia[Title/Abstract]
#14	Atypical Neuralgias[Title/Abstract]
#15	Iliohypogastric Nerve Neuralgia[Title/Abstract]
#16	Iliohypogastric Nerve Neuralgias[Title/Abstract]
#17	Ilioinguinal Neuralgia[Title/Abstract]
#18	Ilioinguinal Neuralgias[Title/Abstract]
#19	Nerve Neuralgia, Iliohypogastric[Title/Abstract]
#20	Nerve Neuralgias, Iliohypogastric[Title/Abstract]
#21	Nerve Pain, Paroxysmal[Title/Abstract]
#22	Nerve Pains[Title/Abstract]
#23	Nerve Pains, Paroxysmal[Title/Abstract]
#24	Neuralgias[Title/Abstract]
#25	Neuralgias, Atypical[Title/Abstract]
#26	Neuralgias, Iliohypogastric Nerve[Title/Abstract]
#27	Neuralgias, Ilioinguinal[Title/Abstract]
#28	Neuralgias, Perineal[Title/Abstract]
#29	Neuralgias, Stump[Title/Abstract]
#30	Neuralgias, Supraorbital[Title/Abstract]
#31	Neuralgias, Vidian[Title/Abstract]
#32	Neurodynias[Title/Abstract]
#33	Neuropathic Pains[Title/Abstract]
#34	Pain, Nerve[Title/Abstract]
#35	Pain, Neuropathic[Title/Abstract]
#36	Pain, Paroxysmal Nerve[Title/Abstract]
#37	Pains, Nerve[Title/Abstract]
#38	Pains, Neuropathic[Title/Abstract]
#39	Pains, Paroxysmal Nerve[Title/Abstract]
#40	Paroxysmal Nerve Pains[Title/Abstract]
#41	Perineal Neuralgia[Title/Abstract]
#42	Perineal Neuralgias[Title/Abstract]
#43	Stump Neuralgia[Title/Abstract]
#44	Stump Neuralgias[Title/Abstract]
#45	Supraorbital Neuralgia[Title/Abstract]
#46	Supraorbital Neuralgias[Title/Abstract]
#47	Vidian Neuralgia[Title/Abstract]
#48	Vidian Neuralgias[Title/Abstract]
#49	or/1–51
#50	Long non-coding RNA[Title/Abstract]
#51	LncRNA[Title/Abstract]
#52	or/52–53
#53	#49 and #52

### 2.3 Inclusion criteria for study selection

The included articles must meet the following inclusion criteria:

(1) Case-control studies assessing the association between the LncRNAs expression level and NC.
(2) Patients diagnosed with NC in the case group and healthy people in the control group.
(3) Detailed LncRNAs expression level data provided.
(4) If serial studies from the same group of people were reported, including the latest study.
(5) The search was not limited to the language or date publication.

The criteria for excluding literature are summarized as follows:

(1) Non-human experiments were carried out.
(2) Repeatedly published literature.
(3) The lack of adequate information to calculate the statistical index standard mean difference.

### 2.4 Data collection and analysis

#### 2.4.1 Selection of studies

**Figure 1:**
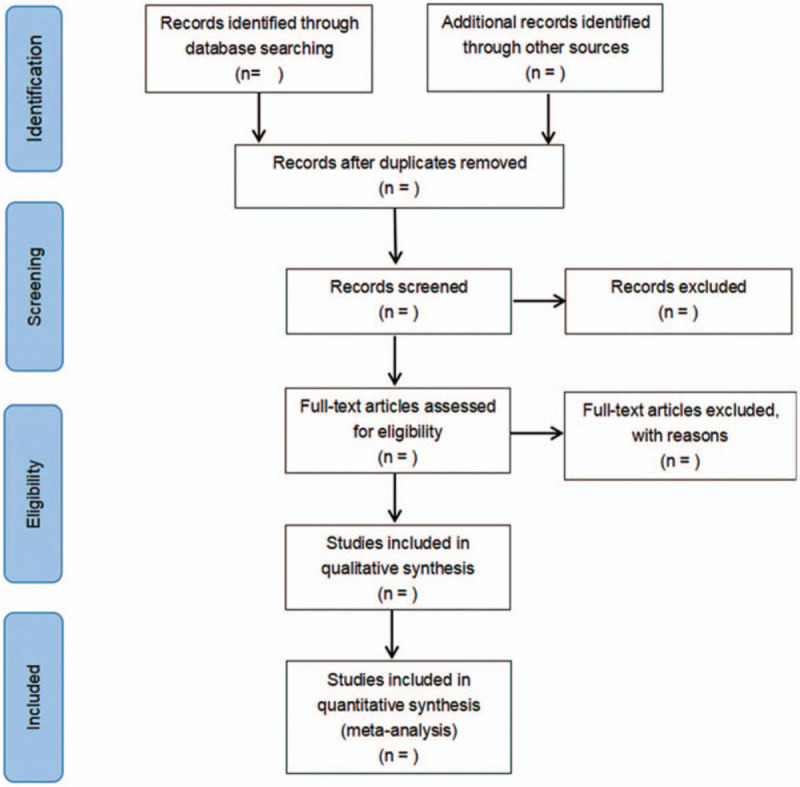
Flow diagram of study selection process.

All reviewers received evidence-based training and adhered to the process that was summarized based on the PRISMA flowchart (Fig. 1). The 2 authors independently screened the literature on the basis of the title, abstract and key words of the literature, and excluded the irrelevant literature. The rest of the literature would further confirm by the 2 authors after reading the full text. The excluded research and the reasons for the exclusion were record. The existing dispute was settled by the third author.

#### 2.4.2 Data extraction and management

Two authors independently extracted the data from the eligible studies. Data entry was conducted using the EpiData software (version 3.0; The EpiData Association, Odense, Denmark). Disagreements were resolved by discussion or consensus with a third author.

If the LncRNAs expression level data was not directly reported, data was extracted from the statistical graph using Engauge Digitizer version 4.1 (Http://digitizer.sourceforge.net/). The following information was extracted: the first author, publishing year, country, ethnicity, specimen, detection method, sample size, etc.

### 2.5 Assessment of quality in included studies

The quality of all the included studies was evaluated by 2 authors independently based on the Newcastle-Ottawa scale that was used to evaluate the quality of observational studies.^[20]^ Disagreement were reported and resolved by a third author.

### 2.6 Measures of results

The data included in the study was continuous, standardized mean difference and 95% confidence interval analysis.

### 2.7 Management of missing data

If there is insufficient or missing data in the literature, we would contact the author via email to request the data. If the data is not available, we would only analyze the currently available data and discuss its potential impact.

### 2.8 Statistical analysis

Meta-analysis was performed using STATA 14.0 (STATA Corporation, College Station, TX). A random effects model was used to identify the effect of each LncRNAs. The results were all presented as logORs, based on the numbers of dysregulation events in NP groups and control samples, with their 95%CI. Compared with control group, logOR values higher than 1 indicated LncRNAs up-regulation. The results were considered statistically significant for *P* values < .05. Prediction of LncRNAs targets was performed by Long Noncoding RNA Database v2.0, the LncRNA and Disease Database, Linc2GO Database and LNCipedia Database, and bioinformatic analysis including Gene Ontology and Kyoto Encyclopedia of Genes and Genomes pathway analysis were performed by KOBAS 3.0. The −log10 (corrected *P*-Value) yields an enrichment score.

### 2.9 Additional analysis

#### 2.9.1 Subgroup analysis

We conducted a subgroup analysis based on the sample type and detection method.

#### 2.9.2 Sensitivity analysis

Sensitivity analysis was performed on the sample size.

#### 2.9.3 Reporting bias

If the number of studies included in a certain outcome index is no less than 10, funnel chart is used to evaluate publication bias.^[21,22]^

### 2.10 Ethics and dissemination

It is not applicable for this systematic review and meta-analysis to require an ethical approval, because this study is not involved with individual patient data. Besides, this review would be disseminated in peer-review journals.

## 3 Discussion

NP is a kind of pain caused by injury or disease of the somatosensory system.^[23–25]^ In the general population, the prevalence rate of NP is 6.9% Murray 10.0%, which seriously perplexes the life quality of patients and brings great burden to individuals, families and society.^[26,27]^ Many factors, including injury or congenital disease,^[28,29]^ can lead to neuropathic pain. Therefore, it is of great significance to explore the molecular targets of NP therapy. In recent years, scholars have explored a new way of targeted treatment of NP by regulating LncRNAs.

Li et al discovered that IncRNA MRAK009713 plays an important regulatory role in the pain model of rats with chronic compressive injury.^[30]^ MRAK009713 can promote pain by regulating the expression and function of P2X3 receptor. IncRNAs aggravates NP through promoting the release of pro-inflammatory factors or activating the phosphorylation of extracellular regulated protein kinases.^[31]^ IncRNAs induces spontaneous abnormal electrical activity and hyperalgesia via regulating the expression of voltage-gated channels.^[32]^ IncRNAs indirectly affects pain by regulating the expression of NP-related microRNA.^[33]^ The above research implies the great prospect of the application of IncRNA in NP. However, the relationship between the expression of LncRNAs and NP remains unclear, which does not take the advantages of the further development of future researches.

Therefore, there is an urgent need for a systematic review ofthe research on the relationship between the expression of LncRNAs and NP. This paper forms our system review scheme, which describes the implementation of the review in detail. The results of our review will be reported in strict accordance with PRISMA standards. By integrating the previous literature, this review objectively reveals the relationship between the expression of LncRNAs and NP. The results of this upcoming study will help us to understand the relationship between the expression of LncRNAs and NP, and will provide a reference for targeting new approaches to the treatment of NP.

The advantages of this study include following aspects: We include the latest literature. For the exploration of heterogeneity, we try to avoid post-group subgroup analysis. In order to improve the credibility of the results, we conduct sensitivity analysis. in addition, we will screen out different LncRNAs to point out the direction for future researches.

In summary, this study will provide up-to-date evidence support for the relationship between LncRNAs expression and NP, and provide a new strategy for targeted therapy of NP.

## Author contributions

**Conceptualization:** Xianbao Yao.

**Data curation**: Yongzhi Fan and Na Li.

**Formal analysis**: Yongzhi Fan and Na Li.

**Funding acquisition:** Xianbao Yao.

**Methodology**: Yongzhi Fan and Na Li.

**Project administration**: Xianbao Yao.

**Resources:** Yongzhi Fan, Na Li.

**Software:** Na Li.

**Supervision**: Xianbao Yao, Na Li.

**Validation**: Xianbao Yao, Na Li.

**Visualization and software**: Yongzhi Fan and Na Li.

**Visualization:** Na Li.

**Writing – original draft**: Yongzhi Fan, Na Li and Xianbao Yao.

**Writing – review & editing:** Xianbao Yao, Yongzhi Fan, Na Li.
